# Supplementary material for: Ecological processes underlying the emergence of novel enzootic cycles: Arboviruses in the neotropics as a case study
Source: PLoS Negl Trop Dis. 2020 Aug 13;14(8):e0008338. doi: 10.1371/journal.pntd.0008338 (PMC7425862; doi:10.1371/journal.pntd.0008338)
Supplement: S1 Text — (DOCX) [file pntd.0008338.s001.docx]

# Supporting Information

***Simulation model methods***

Our approach builds on Althouse et al. (1), which explored how the force of infection and primate birthrate affect the probability that ZIKV will establish a sylvatic cycle in the Americas. Expanding their analysis, we explored the effects of the mosquito extrinsic incubation period (EIP) and lifespan, and primate birthrate, and considered DENV, CHIKV, and YFV in addition to ZIKV. We simulate the introduction of a single infected primate into metapopulations of susceptible primate hosts and mosquito vectors, applying the Gillespie stochastic simulation algorithm with the Binomial Tau Leap approximation to the following transition rates for mosquitoes:


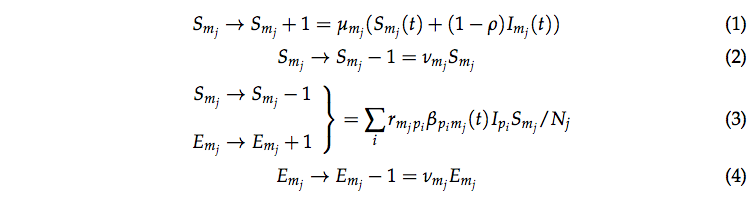


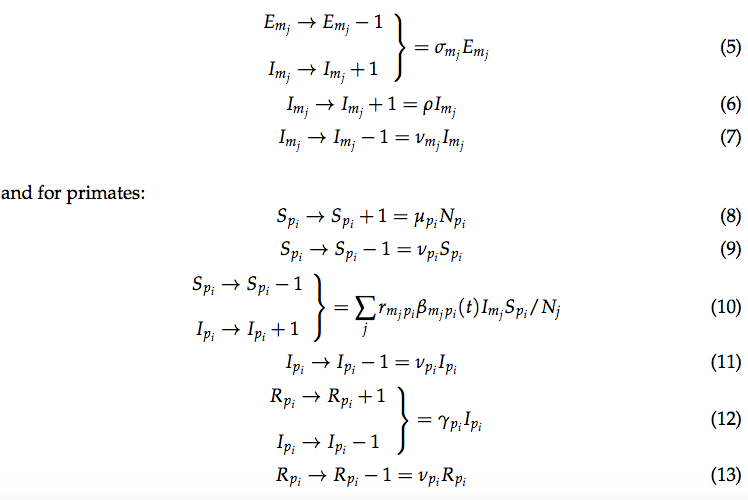


The equations divide primate and mosquito metapopulations into primate species 1,...i and mosquito species 1,...j. The model assumes host preference, with mosquito species j biting the corresponding primate species j more often than other primate species—characterized by the on-diagonal rates within a contact matrix. Primate-mosquito species pairs are coupled by off-diagonal cross-biting rates, calculated as a fixed fraction (10%) of the on-diagonal within-pair biting rates. Given that vertical transmission in the mosquito population is thought to be negligible in enzootic cycles (2), the mosquito transovarial transmission rate $\rho$ is set to zero. Thus, all primates and mosquitoes are born susceptible to arbovirus infection at rates $\mu$ and infected at rates $\beta$(t), proportional to the number of mosquito bites given or received per day and the probability of successful transmission. We assume that birthrate = 1/lifespan—a conservative estimate given that primates’ peak reproductive years occur before the age of mortality (3) and mosquitoes have the capacity to complete multiple gonotrophic cycles within a lifetime (4). Thus, birthrates vary as we explore three mosquito lifespans (7, 14, and 21 days). Transmission probabilities vary seasonally in Equation 14 and 15 due to changes in environmental conditions such as rainfall and temperature (5). After infection, mosquitoes enter the exposed compartment, progressing to the infectious period at one of three fixed rates $\sigma_{m_{j}}$ (1/2, 1/7, or 1/10 days).

Primates become infectious immediately and recover at a fixed rate $\gamma_{p_{i}}$, whereas mosquitoes remain infectious for life. Within each simulation, all parameter values are held constant across mosquito and primate species. Between simulations, only the EIP, and mosquito and primate lifespans, and number of species are varied—all other parameter values are fixed. Holding parameters constant whenever possible allowed us to minimize model complexity and target the effects of our parameters of interest. Furthermore, for the majority of these parameters, there is not enough empirical data to accurately characterize differences between New World species. Full lists of parameter values used for the simulations presented in Figure 2 are given in the table below. For each set of parameter values, we ran 50 simulations and calculated the probability of sylvatic establishment as the proportion of simulations in which there were infected primates remaining at the end of the simulated three-year period.


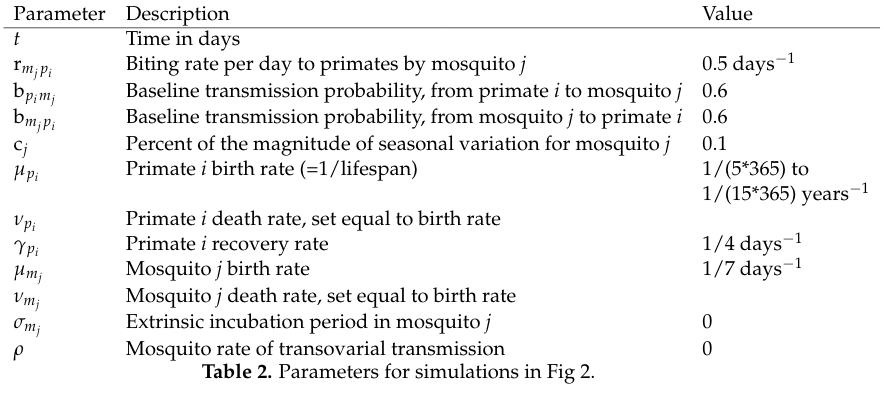


By default, the compartmental model described in Equation 1 through 13 assumes exponentially distributed waiting periods. This assumption is biologically unrealistic because individuals leave their current compartment at a constant rate, regardless of how much time has passed since they entered that compartment. For example, with constant rates, individuals have an equal probability of leaving the latent compartment regardless of the time since infection. In reality, the probability of progressing should be very low immediately after infection, and highest around the latent period mean—a trajectory better represented by a gamma distribution, or it’s discrete analog, the Erlang distribution. The use of exponentially distributed infectious and latent periods significantly affects model estimations of the basic reproductive number, duration of epidemics, and critical population size required for disease persistence in directly transmitted pathogens (6–8). Given the short mosquito lifespan and the resulting interaction between EIP and the length of the infectious period, epidemic dynamics would be more sensitive to the distribution of waiting periods in the mosquito vector than in the primate host. Thus, we explored the effect of modeling the latent period in the mosquito vector (EIP) as an Erlang distribution by splitting the latent period into a series of separate compartments, commonly referred to as a boxcar configuration (9).

We constructed two models with Erlang-distributed EIPs—a model with 10 exposed compartments, and a model with 50 exposed compartments. Figure S1 demonstrates the differences between the probability distributions of the exponentially-distributed EIP relative to both Erlang-distributed EIPs. As expected, modeling EIP as less-dispersed Erlang distributions resulted in higher probabilities of longer EIPs, thus decreasing the probability of sylvatic persistence. However, the Erlang-distributed models produced the same general trends as the exponentially distributed models, with shorter EIPs and longer mosquito lifespans increasing the probability of sylvatic establishment. As a result, in the interest of minimizing model complexity, in our main text, we report results from our exponentially-distributed model. Simulation results from the Erlang-distributed models with respect to differences in the mosquito EIP and lifespan are presented in Figure S2 and S3.

**References**

1. Althouse BM, Vasilakis N, Sall AA, Diallo M, Weaver SC, Hanley KA. Potential for Zika Virus to Establish a Sylvatic Transmission Cycle in the Americas. PLOS Neglected Tropical Diseases. 2016 Dec 15;10(12):e0005055.

2. Adams B, Boots M. How important is vertical transmission in mosquitoes for the persistence of dengue? Insights from a mathematical model. Epidemics. 2010 Mar 1;2(1):1–10.

3. Alberts SC, Altmann J, Brockman DK, Cords M, Fedigan LM, Pusey A, et al. Reproductive aging patterns in primates reveal that humans are distinct. Proc Natl Acad Sci U S A. 2013 Aug 13;110(33):13440–5.

4. Briegel H. Physiological bases of mosquito ecology. J Vector Ecol. 2003 Jun;28(1):1–11.

5. Althouse Benjamin M., Hanley Kathryn A. The tortoise or the hare? Impacts of within-host dynamics on transmission success of arthropod-borne viruses. Philosophical Transactions of the Royal Society B: Biological Sciences. 2015 Aug 19;370(1675):20140299.

6. Keeling MJ, Grenfell BT. Effect of variability in infection period on the persistence and spatial spread of infectious diseases. Mathematical Biosciences. 1998 Jan 15;147(2):207–26.

7. Lloyd AL. Realistic Distributions of Infectious Periods in Epidemic Models: Changing Patterns of Persistence and Dynamics. Theoretical Population Biology. 2001 Aug 1;60(1):59–71.

8. Wearing HJ, Rohani P, Keeling MJ. Appropriate Models for the Management of Infectious Diseases. PLOS Medicine. 2005 Jul 26;2(7):e174.

9. Getz WM, Dougherty ER. Discrete stochastic analogs of Erlang epidemic models. Journal of Biological Dynamics. 2018 Jan 1;12(1):16–38.
