# Supplementary figures and images for: Ecological processes underlying the emergence of novel enzootic cycles: Arboviruses in the neotropics as a case study
Source: PLoS Negl Trop Dis. 2020 Aug 13;14(8):e0008338. doi: 10.1371/journal.pntd.0008338 (PMC7425862; doi:10.1371/journal.pntd.0008338)

**EIP = 2 days**

Probability of progressing  
to infectious

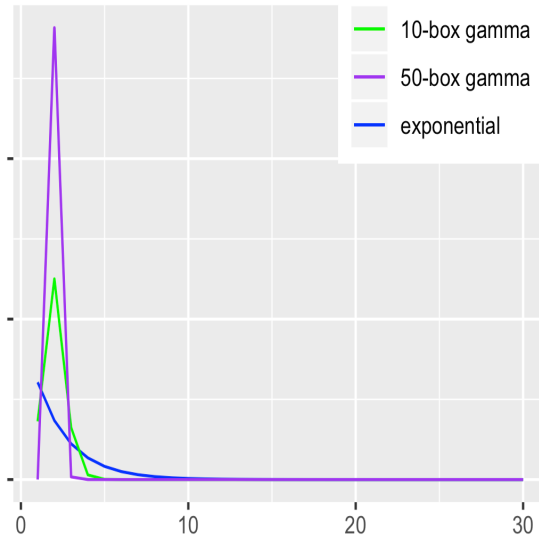

**EIP = 7 days**

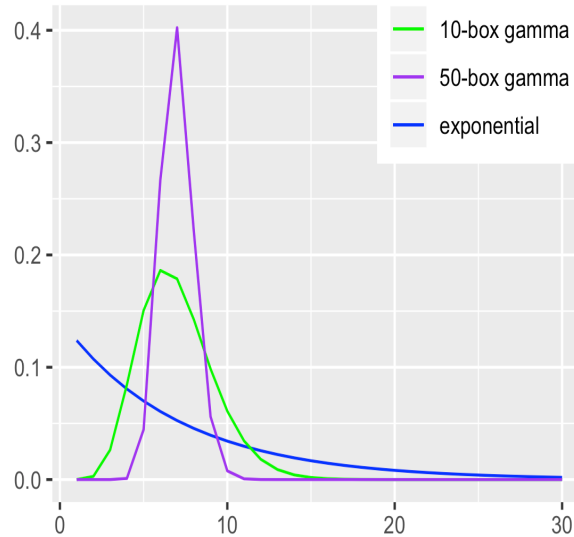

**EIP = 10 days**

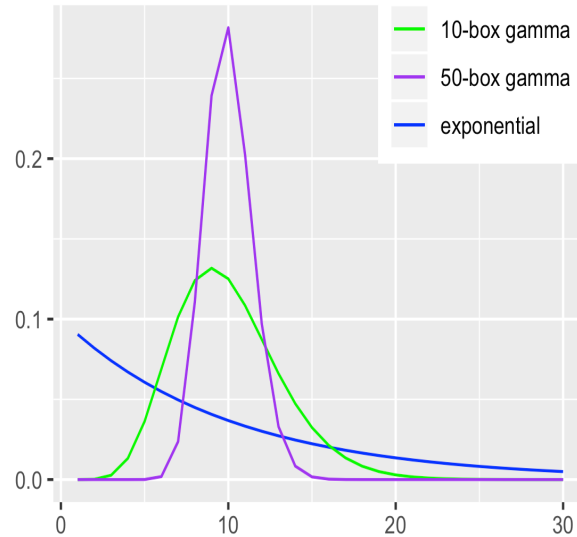

time (days)

Supplement: S1 Fig — In the case of EIP, this assumption is unrealistic because individuals have an equal probability of leaving the incubation compartment regardless of the time since infection. In reality, the probability of progressing should be very low immediately after infection and highest around the EIP mean—a trajectory better represented by a gamma distribution (in green and purple). A gamma distributed EIP can be constructed in a compartmental model via a boxcar configuration of the latent period (i.e., splitting the latent period up into a series of separate compartments, or boxes). Note how, as the number of latent boxes increases (green versus purple), the gamma distribution becomes less dispersed and more closely centered around the EIP mean. EIP, extrinsic incubation period. (PDF) [file pntd.0008338.s002.pdf]
