# Supplementary material for: Ecological processes underlying the emergence of novel enzootic cycles: Arboviruses in the neotropics as a case study
Source: PLoS Negl Trop Dis. 2020 Aug 13;14(8):e0008338. doi: 10.1371/journal.pntd.0008338 (PMC7425862; doi:10.1371/journal.pntd.0008338)
Supplement: S2 Fig — Mosquito birthrate = 1/life span and increases from the top to bottom panels, while EIP increases left to right. Here, we constructed an Erlang-distributed EIP by splitting the exposed compartment into 10 separate boxes. Within each panel, the total population size of mosquitoes (in two populations) and primates (in two populations) changes horizontally and vertically, respectively. For each parameter set, we simulated the introduction of a single infected primate and subsequent transmission for a three-year period. Blue indicates no simulations establishing, whereas red indicates all simulations establishing. Contour lines show 0.25, 0.5, 0.75, and 0.95 probability of establishment. EIP, extrinsic incubation period. (PPTX) [file pntd.0008338.s003.pptx]

## Slide 1
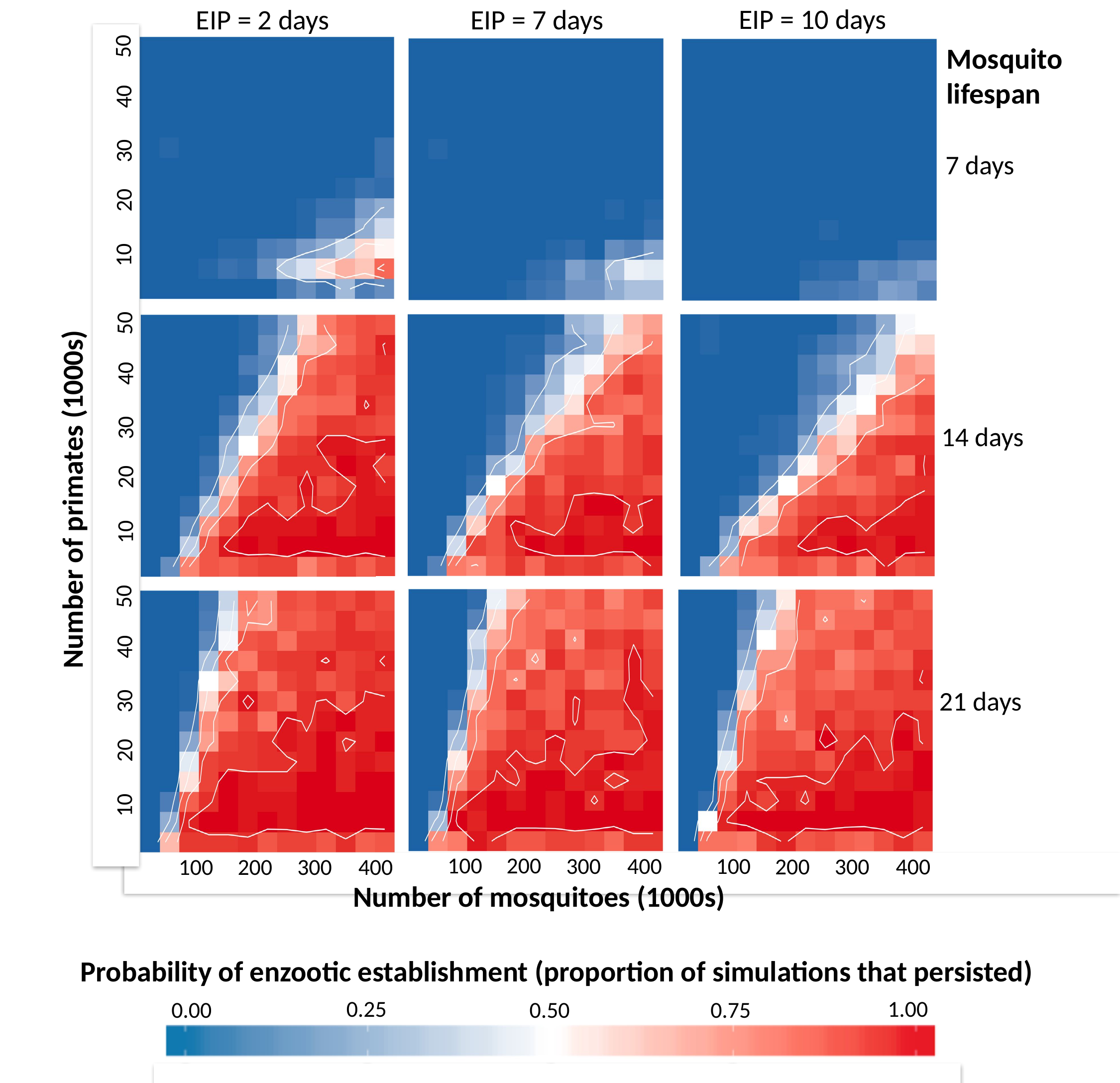

EIP = 10 days
EIP = 2 days
EIP = 7 days
50
Mosquito lifespan
40
30
7 days
20
10
50
40
30
14 days
20
Number of primates (1000s)
10
50
40
21 days
30
20
10
100
200
300
400
100
200
300
400
100
200
300
400
Number of mosquitoes (1000s)
Probability of enzootic establishment (proportion of simulations that persisted)
0.25
1.00
0.50
0.75
0.00
